# A Detailed Correlation of Oral-Health-Related Quality of Life of Patients Undergoing Fixed Orthodontic Therapy

**DOI:** 10.7759/cureus.33854

**Published:** 2023-01-17

**Authors:** Mohammad Raji Alrwuili, Feras Jamal Alwaznah, Rais Ahmed, Sheeba Anwar, Feras Abdulrazzaq Shaikh Omar, Ebtihal Hadi Tairan

**Affiliations:** 1 Department of Orthodontics and Dentofacial Orthopedics, Qurayyat Specialized Dental Center, Qurayyat, SAU; 2 Department of Orthodontics, Government Dental College and Hospital (GDCH), Mumbai, IND; 3 Department of Dentistry, Qurayyat Specialized Dental Center, Qurayyat, SAU; 4 Department of Internal Medicine, Saudi German Hospital, Jeddah, SAU

**Keywords:** health-related quality of life, oral-health-related quality of life, meta-analysis, review, quality of life

## Abstract

Malocclusion is a dental condition that can affect both children's and adolescents’ oral-health-related quality of life (OHRQoL), and the seriousness of the condition is indicated by the patient's requirement for orthodontic therapy. The patient or his or her caretaker may personally report the necessity for orthodontic therapy, or the doctor or orthodontist may quantify it objectively. However, discrepancies in the requirement for either objective orthodontic therapy or subjective orthodontic therapy have been noted. The OHRQoL measurements should be used in conjunction with the indicator of orthodontic therapy requirement to represent the patient's anticipated treatment requirement. Some systematic reviews have revealed evidence that malocclusion has a detrimental effect on OHRQoL. In addition to the effects of malocclusion, the impact of orthodontic therapy on OHRQoL has also been documented.

There is a dearth of related follow-up studies, particularly those documenting OHRQoL improvements in adolescents both before the beginning of treatment and termination of orthodontics treatment. Additionally, it has been hypothesized that self-esteem affects OHRQoL, albeit there is insufficient data to support either its specific function or its connection to perceptions of oral health. As a result, the purpose of this literature review is to determine whether patients receiving fixed orthodontic therapy report any changes in their reported OHRQoL. There was an extensive review of available original research, case reports, systematic reviews, literature reviews, etc., available in reliable sources of information like PubMed, Scopus, Web of Science, etc. The review found that the process of receiving orthodontic therapy might be unpleasant, affecting OHRQoL. The discomfort caused by orthodontic equipment, which are foreign things put into a delicate portion of the body, is both psychological and physical. Such discomfort may have a detrimental effect on the patient's willingness to receive therapy, their participation, and the treatment's effectiveness. The main sources of discomfort that patients undergoing fixed orthodontic treatment report are the appliance's design, amount of force used in the early stages of their therapy, prior painful memories, emotional variables, cognitive variables, and environmental factors such as age, sex, and culture. As a result, orthodontic treatment may have negative effects on a person's QoL that, in most situations, are temporary.

## Introduction and background

The World Health Organization (WHO) labels quality of life (QoL) as "the individuals personal view of their place in life in accordance with the objectives, aspirations, norms, and worries in the framework of the society and value systems under which they live" [1]. The term *oral-health-related QoL* (OHRQoL) refers to the impact that oral and dental diseases have on a person's QoL [1-4]. Identification of the functional, psychological, and social effects of malocclusion depends on OHRQoL measures [5-7]. The process of receiving orthodontic therapy might be unpleasant, affecting OHRQoL. The discomfort caused by orthodontic equipment, which are foreign things put into a delicate portion of the body, is both psychological and physical [8-10]. Such discomfort may have a detrimental effect on the patient's willingness to receive therapy, their participation, and the treatment's effectiveness [11-13].

In addition to the initial speech issues brought on by malocclusion, it has been observed that orthodontic appliances, which are foreign bodies in the mouth, can also create speech issues, having a negative impact on OHRQoL. Orthodontic devices frequently press against the palate and tooth surfaces, which change the tongue's motility and the amount of space in the mouth, distorting some particular sounds and affecting OHRQoL [14-16].

Pain is a primary barrier to receiving orthodontic therapy, a feature that lowers patient compliance while receiving treatment and negatively affects OHRQoL, and a cause of treatment termination or missing an appointment. This field has been unexpectedly ignored in previous research, educational systems, and practice despite having a significant clinical significance. Orthodontists frequently underestimate the level of discomfort brought on by therapy and are ill-equipped to determine whether and when a patient might require pain medication. Fewer studies have evaluated pain and OHRQoL. Most of the earlier research had significant errors. Some studies were affected by general flaws in research design or quality, whereas others were constrained by comprehensible experimental constraints. Small sample numbers or one-week-long research has a negative impact on several studies [17,18].

Therefore, this detailed narrative review was carried out to extensively explore the available literature regarding the impact of fixed orthodontic treatment on OHRQoL.

## Review

What is OHRQoL?

Health service researchers have concentrated on health as a multidimensional concept in response to the WHO's definition of health as "a full condition of physical, mental, and social well-being and not merely the absence of sickness" [11]. The biopsychosocial model of health, which incorporates symptoms, physical functioning, and emotional and social well-being, is embraced by this idea of health status. In nearly every aspect of physical and mental healthcare, including oral health, QoL, or people's perceptions of their position in life in the context of culture and value systems in which they live and about their goals, expectations, standards, and concerns is now acknowledged as a valid parameter in patient assessment. The model takes into account how perceptions of oral health and associated QoL are influenced by environmental or contextual variables, including access to care, sociocultural influences, education, and family structure. According to theory, OHRQoL is a function of different symptoms and experiences and reflects the individual's subjective viewpoint.

Discomfort Caused by Fixed Orthodontic Appliances: Causes and Effects on OHRQoL

Longitudinal research by Feu et al. showed that individuals who had already had their braces withdrawn had a greater standard of life than those who were still undergoing orthodontic therapy [19]. The majority of research that addresses factors such as patient collaboration and compatibility with orthodontic therapy concentrates on the orthodontist's point of view; however, little emphasis has been paid to the patient’s perceptions of difficulty. A previous detailed literature review also demonstrates that the majority of investigations on an individual's discomfort during orthodontic therapy focus on pain-related issues. As a result, many other factors relating to the patient’s physical and mental health have not been taken into account. The reality that almost all surveys do not use suitable instruments for determining the precise impact of using an orthodontic device on a patient's standard of living is one potential cause [20].

According to research by Marques et al. [21], 16% of respondents complained of discomfort as a consequence of using a fixed orthodontic device, which had a detrimental effect on their standard of life. Therefore, increasing the likelihood that orthodontic therapy will be successful requires determining the causes of this unpleasantness and knowing how such things influence the physical well-being and psychological well-being of those who have braces. Each patient has unique qualities, including self-esteem, treatment adherence, self-confidence, perception of dental aesthetics, and expectations from the fixed orthodontic treatment, all of which are directly related to their impression and level of discomfort [22].

The presence of discomfort was not affected by self-esteem in a study by Marques et al. [21], although the literature clearly shows that the self-belief of patients may be impacted by speech restrictions and brace visibility, particularly during social encounters when the face and lips are the focus of attention. Notably, teenagers who pursue orthodontic therapy primarily due to how others perceive them feel more frequently in the spotlight among their colleagues and classmates while undergoing therapy. The research by Marques et al. [21] had an intriguing and unexpected conclusion that there was no correlation between both discomfort and pain. Tooth motion generated by orthodontic brackets results in pain, and it has been mentioned that the apprehension of pain is a critical aspect of attempting to prevent individuals from pursuing orthodontic therapy. Contrarily, pain experienced during therapy steadily worsens 4 to 24 hours after the orthodontic appliance has been adjusted but reverts to baseline by the seventh day of therapy. As a result, as the therapy progresses, patients can become accustomed to discomfort and pain as the feelings either vanish altogether or, at the most, stop occupying their thoughts. This could be the reason why participants in this study did not report that pain had a negative impact on their standard of life. Additionally, each subject had been receiving orthodontic care for a minimum of six months [22-25].

This finding may help orthodontists in their clinical practices as it will help to educate potential clients about the potential physical as well as psychological consequences of orthodontic therapy. The force applied by the orthodontic arch, particularly in the beginning stages of therapy, is another factor that may cause pain. Light forces are thought to be more biologically effective and less stressful during the movement of teeth in orthodontic treatment according to traditional histology investigations. Additionally, the potential for higher levels of force increases with the increase in baseline severity of crowding as more teeth will be effectively encompassed by an archwire. The acceptance of and adaptability to the discomfort brought on by fixed orthodontic devices were higher in younger individuals [22]. This result supports observations from earlier studies by Scheurer et al. [24], Jones and Chan [25], and Polat and Karaman [23]. However, it deviates from observations from a different study by Scott et al. [26]. These discrepancies could be attributed to the study of the structure and cultural factors. Poor dental hygiene, speaking difficulties, and tooth instability also had a negative impact on participants’ everyday activities. These results underscore the necessity for efficient communication between patients and orthodontists and emphasize that orthodontists should not ignore the distress experienced by people wearing fixed orthodontic products. Halitosis, caries, and other confounding variables were managed. The inconvenience associated with using fixed appliances was the only dependent variable. The statistical model excluded people who claimed to be uncomfortable without using the appliance and with more than one category of discomfort. To further understand the essential phases of how using the appliance negatively affects people's QoL, more research is required [21,27,28].

Pain and Associated Problems Perceived During the Initial Stage of Active Fixed Orthodontic Treatment and OHRQoL

A patient's social well-being, psychological well-being, aesthetic well-being, functional well-being, and physiological well-being are impacted by orthodontic therapy. Unfortunately, it is also linked to side effects such as iatrogenic harm in debonding of the orthodontic bracket and bonding clearance, resorption of the root apex, caries development, gingival as well as periodontal issues, allergic ulcerations, and circulatory metal buildup. Pain and discomfort are the most frequent and problematic complications of orthodontic therapy. The most severe general pain experienced from a bee sting or hamstring injury is equivalent to the level of orthodontic pain. According to Bergius et al. [27], in the first 24 hours following fixed orthodontic therapy, 87% to 95% of teenagers report feeling pain. Additionally, 39% to 49% of patients report feeling pain before, during, or after having their appliances removed.

As a result, pain is considered the primary barrier to receiving orthodontic therapy, a feature that reduces patient compliance while receiving therapy, and a cause of treatment termination or missing an appointment. This field has been unexpectedly ignored in previous research, educational systems, and practice despite having a significant clinical significance. Orthodontists frequently underestimate the level of discomfort brought on by therapy and are ill-equipped to determine whether and when a patient might require pain medication. Fewer than 10 studies have evaluated pain. All earlier research had significant errors. Some studies were affected by general flaws in research design or quality, whereas others were constrained by comprehensible experimental constraints. Small sample numbers or one-week-long research has a negative impact on several studies [28,29]. Furthermore, as compared to research that looks at the full treatment time or longer, there is debate regarding the incidence and length of orthodontic discomfort described in analyses of short duration. The two most crucial elements of discomfort and pain during orthodontic therapy are its duration and intensity according to Krishnan [30]. Clinical applications of this knowledge include increased patient pleasure and better dental health.

Rakhshan and Rakhshan [31] intended to ascertain the frequency and timing of the discomfort and pain related to fixed orthodontic therapy at various sites and when eating or brushing teeth, given the significance of this generally ignored topic. According to the study's conclusions, minor soreness was the most common side effect of tooth brushing. While eating firm food items, sticky food items, or fibrous foods, all patients had some level of pain or discomfort; however, eating soft foods massively diminished pain. Ninety percent of patients claim that getting braces is a painful job, while 30% may stop getting braces before they should owing to the agonistic pain. Although every study has found that pain exists during orthodontic therapy, there are vital variations in the prevalence estimates, pain levels, and timeframes reported. It is very challenging to quantify pain as it is qualitative by nature and varies widely from instance to instance and between individuals, depending on factors such as age, the amount of force delivered, pain tolerance level, prevailing emotional experience, socioeconomic class, and previous pain experiences, among others. According to the study by Rakhshan and Rakhshan [31], having a relatively high prevalence of pain was in accordance with results from prior studies. In addition to the alveolar soreness, soft tissue diseases and sores brought on by orthodontic devices may also produce pain (Table 1) [28,29].

**Table 1:** Important features of a few studies on correlation of OHRQoL of patients undergoing fixed orthodontic therapy. ERQoL, eating-related quality of life; OHRQoL, oral-health-related quality of life; OHIP-14, Oral Health Impact Profile-14; CPQ, Child Perceptions Questionnaire

Authors (years)	Motive of study	Tools used	Findings
Poude et al. (2020) [8]	To evaluate individuals receiving orthodontic treatment for pain and standard of life associated to dental health	OHLP-14	The fixed orthodontic intervention had at least one adverse effect on the participants' oral health. In order to prevent treatment termination owing to pain, orthodontists should advise patients of potential discomfort.
Babaee Hemmati et al. (2022) [9]	To evaluate patients receiving fixed orthodontic intervention OHRQoL and ERQoL	The OHIP-14 questionnaire and ERQoL questionnaire	The transitory decline in OHRQoL brought on by fixed orthodontic treatment got better with time. With time, the eating issues also became better.
Jawaid et al. (2019) [10]	To assess modifications in OHRQoL 24 hours after the placement of fixed orthodontic devices and analyze the findings	The OHIP questionnaire	Within 24 hours of the separators, bands, and brackets being inserted, OHRQoL drastically decreased. It, however, recovered after some weeks of therapy.
Mansor et al. (2022) [11]	To evaluate changes in patients utilizing fixed orthodontic equipment 24 hours after placement in terms of their OHRQoL	The OHIP-16 questionnaire	Almost all domains of OHRQoL were shown to be worsening within 24 hours of insertion of the fixed orthodontic prosthesis.
Baidas et al. (2020) [12]	To examine the impact of different orthodontic therapies on OHRQoL among Saudi patients who required orthodontic therapy and monitor the alterations in OHRQoL while using orthodontic appliances	The OHIP-14 questionnaire; the OHRQoL negatively correlated with the OHIP-14 score	The study's outcomes can be applied to improve patients' cooperation, anticipation, and commitment to orthodontic therapy in light of the rising rehabilitative and aesthetic requirements of orthodontic therapy and the focus on OHRQoL.
O'Brien et al. (2007) [13]	To evaluate the effects of fixed orthodontic therapies on adults' conscience and OHRQoL	OHIP-14	During the first three months of fixed orthodontic therapy, the OHRQoL as a whole was negatively impacted; however, after that time, OHRQoL returned to pretreatment levels, and as a result of the treatment, a considerable rise in self-confidence is noted.
Brouns et al. (2022) [14]	Preoperative orthodontic therapy influence on OHRQoL particularly estimated in situations of integrated orthodontic and orthognathic intervention in this systematic review	Quality-of-life evaluation	According to studies, OHRQoL declines before the surgical orthodontic intervention but improves following an orthodontic-orthognathic intervention. These conclusions are supported by a small amount of low-quality data.
Jaeken et al. (2019) [15]	To assess the association between the original treatment requirement with alterations in OHRQoL during an orthodontic intervention and the impact of self-respect	OHRQoL questionnaires	After receiving orthodontic treatment, OHRQoL improves.
Sun et al. (2018) [16]	To determine whether categories of OHRQoL may be impacted by various levels of malocclusion in teeth and whether these effects vary	CPQ Orthodontic Treatment Need	According to studies utilizing orthodontic parameters to evaluate malocclusion, children with misaligned teeth may have a functional impairment and a worse social life; children with very significant malocclusion may furthermore endure oral sicknesses and have more negative emotional responses. In the future, it would be helpful to confirm these results using more extensive population-based studies.

Speech Problems in Patients Undergoing Fixed Orthodontic Treatment and OHRQoL

Considering malocclusion has a substantial influence on a patient's standard of life, more and more people are turning to orthodontic therapy. The inability to tolerate orthodontic therapy negatively impacts a patient's satisfaction. Speech problems are one of the most serious complications of orthodontic therapy affecting OHRQoL. Investigations into how orthodontic devices affect speech are important because they can aid orthodontists in comprehending the causes underlying these speech imbalances. This knowledge will help them provide their patients with objective advice about the accompanying speech impairments and the best remedy for these problems.

The impact of orthodontic treatment on speech impairment was discussed in a recent analysis by Doshi and Bhad-Patil [32]. There have even been several research articles comparing the side effects of lingually attached orthodontic devices and labially attached orthodontic devices, and the findings indicated that participants wearing lingually attached appliances experienced higher speech issues. To the greatest of our conscience, however, the effects of various orthodontic devices, including fixed devices on speech impairment have not been thoroughly compiled using a scientific proof approach. In 1956, Feldman [33] said that speech mistakes could occur right away following the placement of a labially attached orthodontic appliance but could be corrected in a matter of weeks. The arrangement of labial devices may result in direct contact seen between labially placed orthodontic brackets and the upper lip in addition to the anterior teeth. Moreover, tongue extrusion from the front part of the mouth may occur, which may interfere with the pronunciation of the sound [34]. The outcomes of the trials that were included show that the longevity of the speech disruption brought on by labially placed appliances varies. The variance may be explained by the various degrees of malocclusion intensity and participants' neural equilibration, which result in variable adaptive capacities [34]. As evidenced by the results of tests carried out by Rai et al. [35], it should also be highlighted that the transpalatal prostheses and lingual reinforcements utilized in conjunction with labially attached appliances can act as the primary cause of a substantial percentage of speech impairment. Due to benefits, including improved aesthetics, a lower risk of cavities, and less anchoring loss, the lingually attached orthodontic device has become more widely used in clinical settings since Fujita, who introduced lingual fixed appliances. Patients using lingually attached appliances may also struggle to keep their mouths clean, feel tongue soreness, and have trouble speaking. Several systematic reviews have confirmed that lingual appliances cause more difficulty speaking than labial appliances [36].

According to Runte et al. [37], the palatally pointed maxillary incisors had an impact on the affricate sound. The geometries of the lingual aspect of the tooth were changed when the orthodontic brackets were positioned on the lingual aspect of teeth of the anterior region of the oral cavity, which caused speech problems. Thus, the bracket layouts and orthodontist approaches may be directly related to speech issues brought on by lingual appliances. According to Sinclair et al. [38], as the tongue came into contact with new lingual prostheses, the frequency range of consonants might be reduced, subsequently lowering the total intensity of words. Additionally, the lingually attached orthodontic brackets' spaces and the structural depth of the appliances may enable air to escape uncontrollably, which makes it difficult to pronounce some consonants like {j}, {d}, and {t} with a seal. When producing vowel sounds, the lingually attached appliances may reduce the volume of the tongue and affect coarticulation [35].

Psychosocial Impacts of Fixed Orthodontic Treatment and OHRQoL

Adults who have dental malocclusions experience negative psychological effects, including difficulty in chewing, pain episodes, psychological distress as a consequence of dental issues, and problems interacting with others, particularly when the malocclusion condition is severe. With oral health, QoL, and the psychosocial effect of maxillofacial aesthetics as the primary research parameters, many studies have evaluated the cognitive advantages of orthodontic therapy. The amount of time coordinates in the aforementioned longitudinal studies varies. Some of them just include only pretherapy evaluations and posttreatment evaluations, while others include six actual measurements over some time [39-41]. Generally, a short-term shift in the effect indicators may be seen, despite the fact that aesthetic worry is not consistent. This is true although the analysis of data may be problematic due to the variety of approaches used. Long-term trends show a considerable decline in all metrics. *The lack of unfavorable implications of conditions of the oral cavity on social life and good perception of dentofacial-associated self-confidence* [42] has been characterized as OHRQoL. QoL associated with orthodontic treatment has been examined across a variety of age groups, varied malocclusion severity grades, and different orthodontic techniques. As a result of the patient-perceived aesthetic enhancement in the arrangement of the anterior teeth, the psychological and social dental burden reduces throughout orthodontic therapy. There has been at least six months since the commencement of the therapy for this progress. The patient's impression of dental-associated social and psychological effects in areas, including confidence in dental procedures, social implications, psychological implications, and aesthetic worry, improves after the fixed orthodontic therapy. Patient personality traits, most of which have been extensively researched in well-being dynamics, such as internal self-discipline and self-competency, may affect the progress of psychological and social effects [43]. The extent to which a person feels capable of successfully regulating one's health outcomes is known as health awareness. To date, very few research studies have linked orthodontics and health competence.

Gazit-Rappaport et al. [44] claimed that the first six months are crucial in terms of the psychological dental impact. Particularly in the first six months of therapy, there is a noticeable improvement in the dental influence variables connected to more subjective components, especially those connected to psychological effects, emotional conditions, and self-esteem. The discrepancies that existed during the first six months of therapy continue but do not widen. This modification resembles how the patient perceives malocclusion. This lessening of psychological effects and a lowering of self-perception can be seen as a result of the malocclusion improving over the first few months of treatment. As this is an early stage, within which 70% to 100% of the total cases of dental overcrowding are corrected, this time appears to play a significant influence in how dental cosmetics are perceived [45,46]. These results support those from the study by Prado et al. [40], who also noted a betterment of psychological aspects. But between baseline and the first six months of therapy, these authors found no appreciable differences in the connection to self-confidence [40]. In terms of visual appearance, a positive shift develops over time. The outcomes of the research by Prado et al. [40] were similar to those of the investigation by González et al. [45] and suggested that the aesthetic appearance deteriorated during six months of therapy but improved at the conclusion. This recovery in psychological impact is attributed to the debonding of the orthodontic appliance and the correction of the misaligned teeth and an enhancement of the expression of a smile [40]. This change was observed by Gazit-Rappaport et al. after the course of treatment and across [44].

Other studies have found detrimental impacts on patients' standard of living in the earlier stages of orthodontic therapy, which is related to the degradation of the sense of aesthetic appeal during orthodontic therapy [40]. The standard of living appears to decline from the beginning of therapy through the first month; this shift appears to gradually recover as time progresses until the completion of therapy and may be related to the inconvenience and initial pain brought on by the fixed orthodontic appliance [31]. Even after the first three months of treatment, this decrease in QoL was still seen, returning to baseline levels at debonding [39]. Irrespective of the style of brackets used, the aforementioned results appear to hold. Regardless of whether traditional orthodontic brackets or self-ligating orthodontic brackets are used, previous studies reveal that there are generally no appreciable increases in QoL. The only variations appear to be in pain sensation, which is less for self-ligating orthodontic brackets, although the variations are not meaningful statistically [44].

Mental Distress in Orthodontic Patients During the COVID-19 Pandemic and OHRQoL

More than 33% of orthodontic respondents stated psychological anguish affecting OHRQoL during the initial COVID-19 pandemic, based on research by Serafim et al. [47]. The form of the orthodontic device, the time elapsed since the last dental appointment, how patients communicated with the dentist, and the areas where the pandemic was progressing, all had an impact on the degree of psychological anguish experienced by orthodontic patients. Nervousness about the length of the therapies was correlated with the form of appliances used. Damaged orthodontic brackets and orthodontic bands, as well as missed deadlines, may affect the length of treatment for both labially fixed and lingual orthodontic prostheses. Because lingual appliances are undetectable, patients appear to be less concerned about the length of the therapy than they are with buccal appliances, which have a comparable duration of treatment. Another factor to take into account is that lingual device users earned more money than buccal appliance users, and patients who are better off financially may be less concerned during this pandemic [48].

When individuals have obtained orthodontic aligners from the orthodontist, they are less influenced by orthodontic catastrophes and missed deadlines and have superior aesthetics than labial appliances. Patients who wear clear aligners consequently tend to be less concerned about the length of therapy. It has been demonstrated that the type of dental facility has a vital impact on the mental behavior of orthodontic patients. While patients undergoing care at orthodontic divisions in dental healthcare hospitals wanted to see their practitioners more desperately after the pandemic, patients enrolled in dental clinics displayed the least individuality to cope up with orthodontic mishaps. These results might be explained by the variations among dental institutes in terms of personnel and facility [49].

## Conclusions

A fixed orthodontic appliance using the patient's social well-being, psychological well-being, aesthetic well-being, functional well-being, physiological well-being, and overall OHRQoL is impacted by orthodontic therapy. The process of receiving orthodontic therapy might be unpleasant. The discomfort caused by orthodontic equipment, which is a foreign thing put into a delicate portion of the body, is both psychological and physical. Such discomfort may have a detrimental effect on the patient's willingness to receive therapy, their participation, and the treatment's effectiveness. The transitory decline in OHRQoL brought on by fixed orthodontic treatment got better with time. Most of the studies found that the fixed orthodontic intervention had at least one adverse effect on the participants' oral-health-associated standard of living. To prevent treatment termination owing to pain, orthodontists should advise patients of potential discomfort. This review outcomes can be applied to improve patients' cooperation, anticipation, and commitment to orthodontic therapy in light of the rising rehabilitative and aesthetic requirements of orthodontic therapy and the focus on OHRQoL.

## References

[1] (2022). WHOQOL: Measuring Quality of Life. https://apps.who.int/iris/handle/10665/63482.

[2] Allen PF (2003). Assessment of oral health related quality of life. Health Qual Life Outcomes.

[3] (2022). Preamble to the Constitution of the World Health Organization as adopted by the International Health Conference, New York; Jun 19-22, 1946. http://whqlibdoc.who.int/hist/official_records/constitution.pdf.

[4] WHOQOL Group (1998). Development of the World Health Organization WHOQOL-BREF quality of life assessment. Psychol Med.

[5] Littlewood SJ, Mitchell L, Lewis BRK, Barber SK, Jenkins FR (2019). An Introduction to Orthodontics. https://global.oup.com/academic/product/an-introduction-to-orthodontics-9780198808664?cc=us&lang=en&.

[6] Proffit WR, Fields HW Jr, Moray LJ (1998). Prevalence of malocclusion and orthodontic treatment need in the United States: estimates from the NHANES III survey. Int J Adult Orthodon Orthognath Surg.

[7] Abu Alhaija ES, Al-Khateeb SN, Al-Nimri KS (2005). Prevalence of malocclusion in 13-15 year-old North Jordanian school children. Community Dent Health.

[8] Poudel P, Dahal S, Thapa VB (2020). Pain and oral health related quality of life among patients undergoing fixed orthodontic treatment: a descriptive cross-sectional study. JNMA J Nepal Med Assoc.

[9] Babaee Hemmati Y, Mirmoayed A, Ghaffari ME, Falahchai M (2022). Eating- and oral health-related quality of life in patients under fixed orthodontic treatment. Clin Exp Dent Res.

[10] Jawaid M, Qadeer TA, Fahim MF (2020). Pain perception of orthodontic treatment - a cross-sectional study. Pak J Med Sci.

[11] Mansor N, Saub R, Othman SA (2012). Changes in the oral health-related quality of life 24 h following insertion of fixed orthodontic appliances. J Orthod Sci.

[12] Baidas LF, AlJunaydil N, Demyati M, Sheryei RA (2020). Fixed orthodontic appliance impact on oral health-related quality of life during initial stages of treatment. Niger J Clin Pract.

[13] O'Brien C, Benson PE, Marshman Z (2007). Evaluation of a quality of life measure for children with malocclusion. J Orthod.

[14] Brouns VE, de Waal AM, Bronkhorst EM, Kuijpers-Jagtman AM, Ongkosuwito EM (2022). Oral health-related quality of life before, during, and after orthodontic-orthognathic treatment: a systematic review and meta-analysis. Clin Oral Investig.

[15] Jaeken K, Cadenas de Llano-Pérula M, Lemiere J, Verdonck A, Fieuws S, Willems G (2019). Reported changes in oral health-related quality of life in children and adolescents before, during, and after orthodontic treatment: a longitudinal study. Eur J Orthod.

[16] Sun L, Wong HM, McGrath CP (2018). Association between the severity of malocclusion, assessed by occlusal indices, and Oral Health related quality of life: a systematic review and meta-analysis. Oral Health Prev Dent.

[17] Stewart FN, Kerr WJ, Taylor PJ (1997). Appliance wear: the patient's point of view. Eur J Orthod.

[18] Liu Z, McGrath C, Hägg U (2009). The impact of malocclusion/orthodontic treatment need on the quality of life. A systematic review. Angle Orthod.

[19] Feu D, Miguel JA, Celeste RK, Oliveira BH (2013). Effect of orthodontic treatment on oral health-related quality of life. Angle Orthod.

[20] Wiechmann D, Gerss J, Stamm T, Hohoff A (2008). Prediction of oral discomfort and dysfunction in lingual orthodontics: a preliminary report. Am J Orthod Dentofacial Orthop.

[21] Marques LS, Paiva SM, Vieira-Andrade RG, Pereira LJ, Ramos-Jorge ML (2014). Discomfort associated with fixed orthodontic appliances: determinant factors and influence on quality of life. Dental Press J Orthod.

[22] Albino JE, Lawrence SD, Tedesco LA (1994). Psychological and social effects of orthodontic treatment. J Behav Med.

[23] Polat O, Karaman AI (2005). Pain control during fixed orthodontic appliance therapy. Angle Orthod.

[24] Scheurer PA, Firestone AR, Bürgin WB (1996). Perception of pain as a result of orthodontic treatment with fixed appliances. Eur J Orthod.

[25] Jones M, Chan C (1992). The pain and discomfort experienced during orthodontic treatment: a randomized controlled clinical trial of two initial aligning archwires. Am J Orthod Dentofacial Orthop.

[26] Scott P, Sherriff M, Dibiase AT, Cobourne MT (2008). Perception of discomfort during initial orthodontic tooth alignment using a self-ligating or conventional bracket system: a randomized clinical trial. Eur J Orthod.

[27] Bergius M, Berggren U, Kiliaridis S (2002). Experience of pain during an orthodontic procedure. Eur J Oral Sci.

[28] Erdinç AM, Dinçer B (2004). Perception of pain during orthodontic treatment with fixed appliances. Eur J Orthod.

[29] Fujita K (1979). New orthodontic treatment with lingual bracket mushroom arch wire appliance. Am J Orthod.

[30] Krishnan V (2007). Orthodontic pain: from causes to management--a review. Eur J Orthod.

[31] Rakhshan H, Rakhshan V (2015). Pain and discomfort perceived during the initial stage of active fixed orthodontic treatment. Saudi Dent J.

[32] Doshi UH, Bhad-Patil WA (2011). Speech defect and orthodontics: a contemporary review. Orthodontics (Chic.).

[33] Feldman EW (1956). Speech articulation problems associated with placement of orthodontic appliances. J Speech Hear Disord.

[34] Paley JS, Cisneros GJ, Nicolay OF, LeBlanc EM (2016). Effects of fixed labial orthodontic appliances on speech sound production. Angle Orthod.

[35] Rai AK, Rozario JE, Ganeshkar SV (2014). Comparison of speech performance in labial and lingual orthodontic patients: a prospective study. Dent Res J (Isfahan).

[36] Long H, Zhou Y, Pyakurel U (2013). Comparison of adverse effects between lingual and labial orthodontic treatment. Angle Orthod.

[37] Runte C, Lawerino M, Dirksen D, Bollmann F, Lamprecht-Dinnesen A, Seifert E (2001). The influence of maxillary central incisor position in complete dentures on /s/ sound production. J Prosthet Dent.

[38] Sinclair PM, Cannito MF, Goates LJ, Solomos LF, Alexander CM (1986). Patient responses to lingual appliances. J Clin Orthod.

[39] Johal A, Alyaqoobi I, Patel R, Cox S (2015). The impact of orthodontic treatment on quality of life and self-esteem in adult patients. Eur J Orthod.

[40] Prado RF, Ramos-Jorge J, Marques LS, de Paiva SM, Melgaço CA, Pazzini CA (2016). Prospective evaluation of the psychosocial impact of the first 6 months of orthodontic treatment with fixed appliance among young adults. Angle Orthod.

[41] Chen M, Wang DW, Wu LP (2010). Fixed orthodontic appliance therapy and its impact on oral health-related quality of life in Chinese patients. Angle Orthod.

[42] Zhou Y, Zheng M, Lin J, Wang Y, Ni ZY (2014). Self-ligating brackets and their impact on oral health-related quality of life in Chinese adolescence patients: a longitudinal prospective study. Sci World J.

[43] Smith MS, Wallston KA, Smith CA (1995). The development and validation of the perceived health competence scale. Health Educ Res.

[44] Gazit-Rappaport T, Haisraeli-Shalish M, Gazit E (2010). Psychosocial reward of orthodontic treatment in adult patients. Eur J Orthod.

[45] González MJ, Romero M, Peñacoba C (2019). Psychosocial dental impact in adult orthodontic patients: what about health competence?. Health Qual Life Outcomes.

[46] Kolenda J, Fischer-Brandies H, Ciesielski R, Koos B (2016). Oral health-related quality of life after orthodontic treatment for anterior tooth alignment: Association with emotional state and sociodemographic factors. J Orofac Orthop.

[47] Serafim CM, Gurgel Jde A, Tiago CM, Tavarez RR, Maia Filho EM (2015). Clinical efficiency of two sequences of orthodontic wires to correct crowding of the lower anterior teeth. ScientificWorldJournal.

[48] Xiong X, Wu Y, Fang X (2020). Mental distress in orthodontic patients during the coronavirus disease 2019 pandemic. Am J Orthod Dentofacial Orthop.

[49] Mavreas D, Athanasiou AE (2008). Factors affecting the duration of orthodontic treatment: a systematic review. Eur J Orthod.

